# Recombinant Antigens rLipL21, rLoa22, rLipL32 and rLigACon4-8 for Serological Diagnosis of Leptospirosis by Enzyme-Linked Immunosorbent Assays in Dogs

**DOI:** 10.1371/journal.pone.0111367

**Published:** 2014-12-19

**Authors:** Cuilian Ye, Weiwei Yan, Hua Xiang, Hongxuan He, Maosheng Yang, Muhammad Ijaz, Nicodemus Useh, Ching-Lin Hsieh, Patrick L. McDonough, Sean P. McDonough, Hussni Mohamed, Zhibang Yang, Yung-Fu Chang

**Affiliations:** 1 Department of Population Medicine and Diagnostic Sciences, College of Veterinary Medicine, Cornell University, Ithaca, New York, United States of America; 2 Department of Biomedical Sciences, College of Veterinary Medicine, Cornell University, Ithaca, New York, United States of America; 3 School of Pharmacy and Bioengineering, Chongqing University of Technology, Chongqing, China; 4 Department of Pathogenic Biology, School of Basic Medical Sciences, Chongqing Medical University, Chongqing, China; 5 Veterinary Institute, Guangdong Academy of Agriculture Sciences, Guangzhou, China; 6 Institute of Zoology, Chinese Academy of Sciences, Beijing, China; 7 Guizhou Institute of Animal Husbandry and Veterinary Medicine, Guizhou Academy of Agriculture Sciences, Longdongbao, Guiyang, China; Federal University of Pelotas, Brazil

## Abstract

Animal leptospirosis is one of the most common zoonotic diseases in the United States and around the world. In a previous study, we applied four recombinant antigens, rLipL21, rLoa22, rLipL32 and rLigACon4-8 of *Leptospira interrogans* (*L. interrogans*) for the serological diagnosis of equine leptospirosis (Ye et al, Serodiagnosis of equine leptospirosis by ELISA using four recombinant protein markers, Clin. Vaccine. Immunol. 21:478–483). In this study, the same four recombinant antigens were evaluated for their potential to diagnose canine leptospirosis by ELISA. A total of 305 canine sera that were *Leptospira* microscopic agglutination test (MAT)-negative (n = 102) and MAT-positive (n = 203) to 5 serovars (Pomona, Grippotyphosa, Icterohaemorrhagiae, Canicola and Hardjo) were tested. When individual recombinant antigens were used, the sensitivity and specificity of ELISA were 97.5% and 84.3% for rLigACon4-8; 89.7% and 81.4% for rLoa22; 92.6% and 84.3% for rLipL32 and 99.5% and 84.3% for rLipL21, respectively compared to the MAT. The sensitivity and specificity of ELISA were, 92.6% and 91.2% for rLigACon4-8 and rLipL32, 97.5% and 84.3% for rLigACon4-8 and rLipL21, 89.7% and 87.3% for rLigACon4-8 and rLoa22, 89.7% and 87.3% to rLipL21 and rLoa22, 92.6% and 91.2% for rLipL21 and rLipL32 and 89.2% and 94.1% for rLoa22 and rLipL32 when one of the two antigens was test positive. The use of all four antigens in the ELISA assay was found to be sensitive and specific, easy to perform, and agreed with the results of the standard *Leptospira* Microscopic Agglutination test (MAT) for the diagnosis of canine leptospirosis.

## Introduction

Leptospirosis is a serious worldwide zoonotic disease that affects various domestic animals, including dogs [1], [2], [3]. Leptospires are transmitted directly or indirectly, mainly through contact with infected urine, and enter the body through mucous membranes or skin abrasions [1], [2], [4]. Heavy rainfall and flooding are associated risk factors for leptospiral infection [2], [5], [6]. The clinical signs of leptospiral infection in dogs vary from subclinical to minimal clinical disease with mild fever to severe kidney and liver failure and pulmonary hemorrhage [3], [7], [8], [9]. Gautam et al reported that 2,680 samples were seropositive for antibodies against *Leptospira* serovars among 33,119 canine serum samples submitted to a commercial veterinary diagnostic laboratory from 2000 through 2007 in the United States [10].

Dogs serve either as accidental hosts for various pathogenic serovars, such as serovars Grippotyphosa and Pomona, or as maintenance hosts for serovar Canicola [1], [11], [12], [13]. In accidental infections with serovars Icterohaemorrhagiae and Grippotyphosa, dogs may show acute or subacute hepatic and renal failure, respectively [8], [13]. However, the distribution of serovars may vary between different countries; therefore different serovars were used to develop a bacterin in different areas. In Europe, four serovars, Canicola, Icterohaemorrhagiae, Grippotyphosa, and Australis have been used to develop a bacterin [14], whereas in the USA, serovars Canicola, Icterohaemorrhagiae, Grippotyphosa, and Pomona were used for bacterin development [15]. In Germany, serovars Australis, Grippotyphosa and Pomona are the predominant serogroups associated with canine leptospirosis [16]. In Thailand, serovar Autumnalis was the predominant serovar in an outbreak of human leptospirosis [17], [18]. The widespread use of bivalent vaccines containing these serovars and the increased contact between dogs and wildlife reservoirs in expanding suburban environments are likely to result in changes of the prevalent *Leptospira* serovars or the emergence of new serovars in the USA and Europe [10], [19], [20], [21]. Although dogs have been diagnosed with *Leptospira* spp infection by serology, the pathogens have not been isolated from most of these clinical cases [3], [22]. Because of the non-specific clinical signs and variable changes in clinical pathology findings, depending on the stage of infection, multiple methods are usually employed for the diagnosis of canine leptospirosis. Four outer membrane antigens were previously found useful for the serodiagnosis of equine leptospirosis by ELISA [23]. In an attempt to improve the specificity and sensitivity of the indirect ELISA test for diagnosis of canine leptospirosis, we evaluated 4 recombinant antigens (LipL21, Loa22, LipL32 and LigACon4-8). LipL21 is a surface-exposed lipoprotein [24]. Loa22 encodes a lipoprotein with an OmpA domain and it is up-regulated during host infection [25]. LipL32 makes up more than 50% of both the outer membrane subproteome and surfaceome [26]. The Lig proteins, which include LigA, LigB, and LigC, are major components of the leptospiral surface and are also upregulated during infection [27], [28]

## Materials and Methods

### Sera

Canine sera were collected from 2009 to 2012 by the New York State Animal Health Diagnostic Center (AHDC), Cornell University, Ithaca, NY. These serum samples were either positive (n = 203) or negative (n = 102) in the MAT to the following serovars: *L. interrogans* serovar Pomona, *L. kirschneri* serovar Grippotyphosa, *L. interrogans* serovar Icterohaemorrhagiae, *L. interrogans* serovar Canicola or *L. borgpetersenii* serovar Hardjo. Experiments were conducted according to the protocol approved by IACUC (Institutional Animal Care and Use Committee) at Cornell University.

### MAT

MAT was used as the reference method to determine the serum titers using live *L. interrogans* as antigen as previously described [29]. Briefly, serial twofold dilutions of the sera, starting with a dilution of 1: 10, were mixed with an equal volume of viable *Leptospira* strains in a 96 well microtiter plate. After incubation at 30°C for 2 h, the samples were examined for agglutination by dark field microscopy. Titers represent the highest serum dilution showing 50% agglutination of the leptospiral cells in the suspension. MAT titers ≥1∶200 were considered a positive serum sample.

### Cloning, expression, and purification of the four recombinant proteins

The 4 recombinant proteins were purified as previously described [23], [30]. *pLip32L* was cloned into pGEX4T2(GE, USA) and expressed and purified as a GST tagged protein. The GST tag was cut with thrombin (20 U/ml in phosphate buffered saline (PBS), pH 7.3) while the fusion protein was bound to the column by incubating at room temperature for 12 h. LipL21, LigACon4-8 and Loa22 were cloned into pET28 (Invitrogen, USA), expressed and purified as His-sumo-tagged fusion proteins. His-sumo tagged proteins were digested overnight on a Ni-NTA column with sumo-specific protease Ulp-1 at 4°C. Following incubation, the untagged proteins were eluted while the GST and His-sumo tags were retained on the glutathione and Ni-NTA resin respectively. The concentrated, untagged proteins were then subjected to SDS-PAGE to check for purity and stored in -80°C until use.

### Optimization of antigen concentration in ELISA assay

For each antigen, 25, 50, 100, and 200 ng of protein were coated onto different wells and incubated at 4°C overnight. A two fold serial dilution of the test sera was used at 1∶500, 1∶1,000, 1∶2,000, and 1∶4,000. The canine MAT positive and negative sera were employed as positive and negative reaction controls, respectively. A serum titer of 1∶800 was selected as the optimum dilution, based on its OD_630_ in the range 0–1.0. For rLipL21, rLipL32and rLoa22, a protein concentration of 100 ng/well was selected for performing the assay, while 50 ng/well was selected for rligA4-8 protein. These concentrations were selected on the basis of titration for optimum reactivity.

### Enzyme-linked immunosorbent assay (ELISA)

Indirect ELISA was performed as previously described [23], [30] using purified proteins of rLigACon4-8, rLipL32, rLoa22 and rLipL21. Purified proteins were diluted in coating buffer (0.05 M NaHCO_3_/Na_2_CO_3_ buffer, pH 9.6) at optimum concentration established by checkerboard titration. One hundred microliters of the diluted antigen were coated on 96-well microtiter plates (Corning, NY) and incubated at 4°C overnight and then followed by blocking with 1% bovine serum albumin in PBS. Sera were optimally diluted in PBS containing 1% bovine serum albumin and 0.05% Tween 20 and then added to the wells for 1 hour at 37°C. The IgG reactivity was detected with peroxidase-labeled anti-dog IgG (KPL, Inc. ML) and TMB 2-Component microwell peroxidase substrate (KPL, Inc. ML). The plates were read at OD_450_ on a microtiter plate reader (BioTek, VT) after the addition of the same volume of TMB stop solution (KPL, Inc. MD.

### Western blot analysis

Western blot analysis was performed on all canine serum samples as previously described [23], [30] using purified antigens, rLigACon4-8, rLipL32, rLoa22 and rLipL21. Briefly, after the purified recombinant proteins were transferred from the SDS-PAGE separation gel to a nitrocellulose membrane (Schleicher & Schuell Biosciences Inc., New Hampshire), the membranes were blocked and subjected to assay using a 1∶200 dilution of canine test serum as the primary antibody and l: 2,000 dilution of alkaline phosphatase labeled goat anti-dog IgG (KPL, ML, USA) as the secondary antibody. A serum sample that was both MAT and ELISA negative was used as the negative control and an experimental positive serum was used as a positive control.

### Statistical analysis

The ELISA performance was evaluated using the MAT as the reference method (gold standard) [23], [30]. The relative sensitivity, specificity and accuracy of ELISA for the detection of anti-*Leptospira* antibodies in dog sera were determined in comparison to the MAT as follows; Sensitivity  = a/(a+b) ×100; Specificity  = d/(c+d) ×100; Accuracy  =  [(a + d)/(a+b+c+d)) ×100, where a is the number of samples positive by both ELISA and MAT; b is the number of samples positive by MAT but negative by ELISA; c is the number of samples negative by MAT but positive by ELISA; and d is the number of samples negative by both MAT and ELISA [31].

## Results

### Evaluation of ELISA in comparison with MAT and Western blot analysis

**Figure 1:**
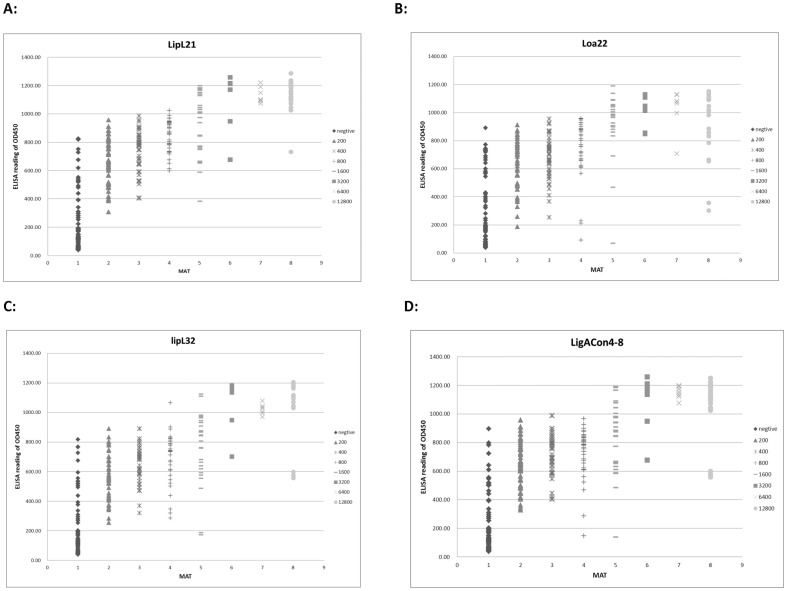
Graph of the ELISA samples showing the IgG ELISA reactivity of 305 canine sera. The *x* axis indicates the MAT titers of the tested sera. The *y* axis indicates the ELISA reading at OD450. A. LipL21; B. Loa22; C, LipL32; D, LigACon4-8.

**Figure 2:**
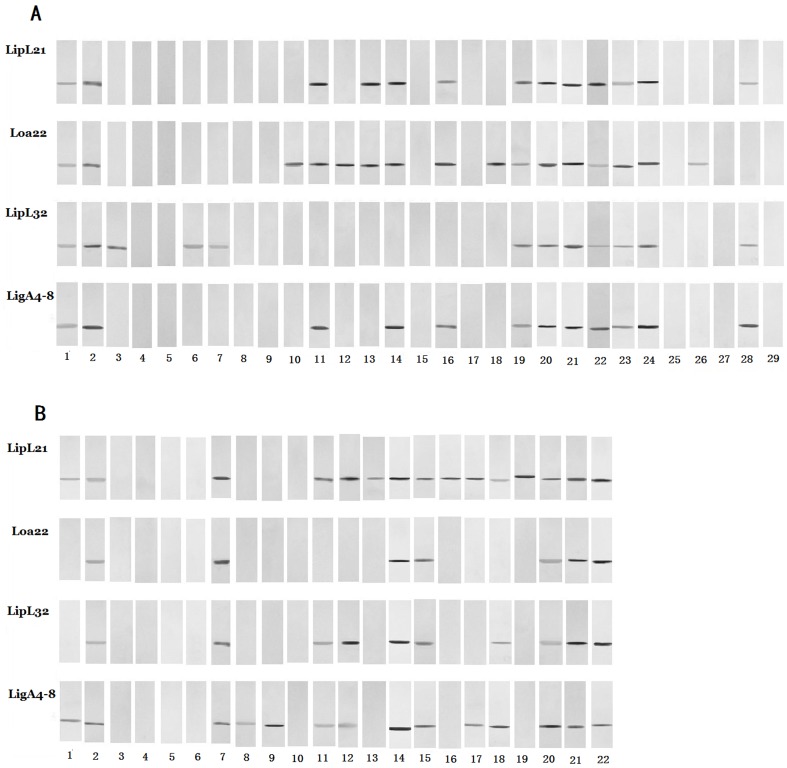
Western blot analysis of canine serum. Sera that were MAT negative but ELISA positive (A) and MAT positive, but ELISA negative (B) were further tested by Western blotting. Purified recombinant proteins rLipL21, rLoa22, rLipL32 and rLigACon4-8 of *L. interrogans* were transferred from the SDS-PAGE separation gel to a nitrocellulose membrane. After washing with TBS, the membranes were blocked and then subjected to assay using the serum to be tested as the primary antibody and l∶3,000-diluted, alkaline phosphatase-labeled goat anti-dog IgG (KPL, Inc., MD) as the secondary antibody. After this, the membranes were incubated in freshly prepared BCIP-NBT color development solution (Invitrogen) for 10 to 30 min to see the results. The number is the dog serum number that was ELISA positive.

For rLipL21, rLipL32and rLoa22, a protein concentration of 100 ng/well was selected for performing the assay, while 50 ng/well was selected for rLigACon4-8 protein. These concentrations were selected on the basis of titration for optimum reactivity. Recombinant proteins rLigACon 4–8, rLipL21, rLipL32 and rLoa22 reacted with MAT positive canine serum samples, and the results are shown in Fig. 1. A, B, C, and D and Table 1. The sensitivity and specificity of ELISA were 97.5% and 84.3% for rLigACon4-8; 89.7% and 81.4% for rLoa22; 92.6% and 84.3% for rLipL32 and 99.5% and 84.3% for rLipL21, respectively compared to MAT (Table 2). When two to four proteins were used and all proteins in each group were ELISA positive, the sensitivity and specificity of ELISA are shown in Table 3., the sensitivity and specificity of ELISA when only one of these proteins was ELISA positive is shown in Table 4. The Western blot analysis of MAT positive and negative samples is shown in Fig. 2. Among MAT negative serum samples, 16, 19, 16 and 16 were ELISA positive to rLipL21, rLoa22, rLipL32 and rLigACon4-8, respectively (Table 5). Among the MAT positive serum samples, 1, 21, 15 and 5 were ELISA negative, but 1, 15, 12, and 3 were Western blot analysis negative to LipL21, LoaL22, LipL32 and LigACon4-8 (Table 5). The ELISA and Western blot analysis of the 29 samples that were MAT negative, but ELISA positive to at least one of these antigens is shown in Table 6. The ELISA and Western blot analysis of the 22 samples that were MAT positive, but ELISA negative to at least one of these antigens is shown in Table 7.

**Table 1:** MAT, ELISA and Western blot analysis of the serum sample used in this study.

protein name	MAT-			MAT+		
	MAT- serum number	MAT- & ELISA-	MAT- & ELISA- & WESTERN-	MAT+ serum number	MAT+ & ELISA+	MAT+ & ELISA+ & WESTERN+
rLipL21	102	86	80	203	202	168
rLoa22	102	83	59	203	182	127
rLipL32	102	86	70	203	188	146
rLigACon4-8	102	86	68	203	198	130

**Table 2:** Sensitivity and specificity of the ELISA test when a single protein was evaluated in comparison to the MAT result.

	sensitivity	specificity
rLipL21	99.5%	84.3%
rLoa22	89.7%	81.4%
rLipL32	92.6%	84.3%
rLigACon4-8	97.5%	84.3%

**Table 3:** Sensitivity and specificity of the ELISA test.

proteins	a*	b*	c*	d*	Sensitivity#	Specificity#
L21&L22	182	21	13	89	89.7%	87.3%
L21&L32	188	15	9	93	92.6%	91.2%
L21&LigA	198	5	16	86	97.5%	84.3%
L22&L32	181	22	6	96	89.2%	94.1%
L22&LigA	182	21	13	89	89.7%	87.3%
L32&LigA	188	15	9	93	92.6%	91.2%
L21&L22&L32	181	22	6	96	89.2%	94.1%
L21&L22&LigA	182	21	13	89	89.7%	87.3%
L21&L32&LigA	188	15	9	93	92.6%	91.2%
L22&L32&LigA	181	22	6	96	89.2%	94.1%
L21&L22&L32&LigA	181	22	6	96	89.2%	94.1%

When all two, three or four of these recombinant proteins were tested positive, the serum sample was judged to be positive. Otherwise, it was judged to be negative.

*a: MAT+&ELISA+; b: MAT+&ELISA-; c: MAT-&ELISA+; d:MAT-&ELISA-;

# Sensitivity  = a/(a+b); specificity  = d/(c+d).

**Table 4:** Sensitivity and specificity of the ELISA test.

proteins	a*	b*	c*	d*	Sensitivity#	Specificity#
L21&L22	202	1	22	80	99.5%	78.4%
L21&L32	202	1	23	79	99.5%	77.5%
L21&LigA	202	1	16	86	99.5%	84.3%
L22&L32	189	14	29	73	93.1%	71.6%
L22&LigA	198	5	22	80	97.5%	78.4%
L32&LigA	198	5	23	79	97.5%	77.5%
L21&L22&L32	202	1	29	73	99.5%	71.6%
L21&L22&LigA	202	1	22	80	99.5%	78.4%
L21&L32&LigA	202	1	23	79	99.5%	77.5%
L22&L32&LigA	198	5	29	73	97.5%	71.6%
L21&L22&L32&LigA	202	1	29	73	99.5%	71.6%

When one of the two, three or four proteins was positive in the ELISA test in comparison to the MAT test, the serum was considered positive.

*a: MAT+&ELISA+; b: MAT+&ELISA-; c: MAT-&ELISA+; d:MAT-&ELISA-;

# Sensitivity  = a/(a+b); specificity  = d/(c+d).

**Table 5:** Comparison of MAT negative and ELISA and Western blot analysis positive or MAT positive, but ELISA and Western blot analysis negative.

	MAT-		MAT+	
Protein name	ELISA+	ELISA+ &WESTERN+	ELISA-	ELISA- &WESTERN-
rLipL21	16	13	1	1
rLoa22	19	14	21	15
rLipL32	16	12	15	12
rLigACon4-8	16	12	5	3

**Table 6:** The ELISA and Western blot analysis of the 29 samples that were MAT negative, but ELISA positive to one to four of these antigens.

Serum #	Lip21L*	Loa22L*	Lip32L*	LipACon4-8*
1	+/+	−/+	+/+	+/+
2	+/+	−/+	+/+	+/+
3	−/−	−/−	+/+	−/−
4	−/−	−/−	+/−	−/−
5	−/−	−/−	+/−	−/−
6	−/−	−/−	+/+	−/−
7	−/−	−/−	+/+	−/−
8	−/−	−/−	+/−	−/−
9	−/−	−/−	+/−	−/−
10	−/−	+/+	−/−	−/−
11	+/+	+/+	−/−	+/+
12	+/−	+/+	−/−	+/−
13	+/+	+/+	−/−	+/−
14	+/+	+/+	−/−	+/+
15	+/−	+/−	−/−	+/−
16	+/+	+/+	−/−	+/+
17	−/−	+/−	−/−	−/−
18	+/−	+/+	−/−	+/−
19	+/+	+/+	+/+	+/+
20	+/+	+/+	+/+	+/+
21	+/+	+/+	+/+	+/+
22	+/+	+/+	+/+	+/+
23	+/+	+/+	+/+	+/+
24	+/+	+/+	+/+	+/+
25	−/−	+/−	−/−	−/−
26	−/−	+/+	−/−	−/−
27	−/−	+/−	−/−	−/−
28	+/+	−/−	+/+	+/+
29	−/−	+/−	−/−	−/−

* ELISA/WB: -, negative; + is positive.

**Table 7:** The ELISA and Western blot analysis of the 22 samples that were MAT positive, but ELISA negative to at least one to four of these antigens.

Serum #	Lip21L*	Loa22L*	Lip32L*	LipACon4-8*
1	+/+	−/−	−/−	+/+
2	+/+	−/+	+/+	+/+
3	−/−	−/−	−/−	−/−
4	+/−	−/−	−/−	+/−
5	+/−	−/−	−/−	+/−
6	+/−	−/−	−/−	+/−
7	+/+	−/+	+/+	+/+
8	+/−	−/−	−/−	−/+
9	+/−	−/−	−/−	+/+
10	+/−	−/−	−/−	+/−
11	+/+	−/−	+/+	+/+
12	+/+	−/−	−/+	+/+
13	+/+	−/−	−/−	+/−
14	+/+	−/+	+/+	+/+
15	+/+	+/+	−/+	+/+
16	+/+	−/−	−/−	−/−
17	+/+	−/−	−/−	−/+
18	+/+	−/−	+/+	+/+
19	+/+	−/−	−/−	−/−
20	+/+	−/+	−/+	+/+
21	+/+	−/+	+/+	+/+
22	+/+	−/+	+/+	+/+

* ELISA/WB: -, negative; + is positive.

## Discussion

The diagnosis of canine Leptospirosis is usually based on direct observation of leptospires in blood or urine samples, the isolation of the pathogens in culture, seropositivity for *Leptospira*-specific antibodies, and/or the demonstration of *Leptospira* DNA by PCR-based assays [32]. The use of these diagnostic techniques for the diagnosis of leptospirosis in dogs has been previously reported [33]. The standard method for diagnosis of leptospirosis is the microscopic agglutination test (MAT), in which serum samples are reacted with live antigen suspensions of *Leptospira* serovars. However, MAT is laborious, time consuming and requires maintaining cultures of the various serovars that are prevalent in some regions but not in others. Thus, considerable efforts are being made to develop novel, sensitive, and specific diagnostic tests for leptospirosis that are less labor and resource intensive. The performance of MAT is restricted only to the laboratories that can maintain strains for the preparation of live antigens. To culture the organism from tissues or body fluids, it is very important to know the stage of infection of the animals; *Leptospira* can only be cultured from blood samples in the acute phase which usually lasts for about 10 days. After the antibody response is detected (at approximately 10 days), *Leptospira* are cleared from the blood. During the second phase, which may last up to several months, bacteriuria is often intermittent, which makes the culture results inconsistent. For the same reason the molecular diagnossis of leptospirosis is only suitable in the early and convalescent stages of infection, although it has been shown to be sensitive and specific. Hence, currently most cases of leptospirosis are still diagnosed by serology. In infected animals, antibodies become detectable by the 6th to 10th day of disease and reach peak levels within three to four weeks. The antibody levels then gradually decline but still can be detected for years [34]. Thus, considerable efforts are being made to develop novel, sensitive, and specific serological diagnostic tests for leptospirosis that are less labor and resource intensive. Enzyme linked immunosorbent assay (ELISA) methods are a potential diagnostic tool for the serodiagnosis of leptospirosis [30], [35], [36]. Attempts have been made to develop either an ELISA serodiagnostic test [31], [37], [38], [39], [40], [41], [42], [43], [44] or a Dual Path Platform (DPP) assay, a point-of-care immunoassay [45]. We previously used the LigA protein for diagnosis of equine and canine leptospirosis [23], [30], [32], [46] and rLigACon4-8, rLipL32, rLoa22 and rLipL21 for diagnosis of equine leptospirosis [23]. We hypothesized that the use of these four antigens in the ELISA test would improve the sensitivity and specificity of this serologic test to canine leptospirosis.

We collected 203 positive plus 102 negative MAT canine sera from 2010 to 2012, for further ELISA evaluation using the four test antigens. The MAT test targets both IgM and IgG, but is skewed towards IgG [1], [47]. Because most of the canine serum samples did not come from an early leptospiral infection, we used rLigConA4-8, rLipL32, rLipL21 and rLoa22 proteins as the coated antigen to establish an ELISA for improved detection of specific IgG in sera from canine patients with positive titers in the MAT test.

A four-fold rise in titer or seroconversion has been used as the definitive criterion for the serologic diagnosis of active leptospirosis. This requires collecting serum samples from the same animal 3 or 4 weeks later and this delay is not practical in the clinical setting. Alternatively, a single high MAT titer may be taken as evidence of active infection. Therefore, the WHO Leptospirosis Burden Epidemiology Reference Group (LERG) and the Centers for Disease Control and Prevention (CDC), US have recently defined a MAT titer of 400 in a single serum specimen as evidence supporting laboratory confirmation [48], [49]. A defined positive titer is also needed in dogs. Cautam, et al. selected ≥1∶1,600 as positive [10]. A MAT ≤400 is not considered indicative of disease attributable to leptospirosis [50]. In Switzerland, a MAT titer ≥800 is defined as positive for clinical canine leptospirosis [51]. However, Andre-Fontaine sets a MAT titer <320 as the cutoff for non-infected, vaccinated dogs [52]. Therefore, further studies are needed to select a universal cut off for the serodiagnosis of canine leptospirosis.

Interestingly, we found that 16, 19, 16, and 16 MAT negative serum samples were positive by ELISA when using rLipL21, rLoa22, rLipL32 and rLigACon4-8 as antigens, respectively. This is not surprising since some of these antigens may be not expressed well by leptospires grown in vitro and MAT would not be able to detect antibody in these serum samples [28], [53], [54]. We further evaluated these MAT negative/ELISA positive serum samples by Western blot analysis, and found that 13/16, 14/19, 12/16 and 12/16 of these respective samples were Western blot analysis positive. This suggests that these dogs were either infected or vaccinated previously but the MAT antibody titers to *Leptospira* lipopolysaccharide antigens declined below the detection threshold (<1∶200). Similarly, some urine culture positive dogs are MAT negative [55]. We did not have animal histories that would have allowed us to ascertain the *Leptospira* vaccination status of the dogs and/or if they exhibited any clinical signs of leptospirosis.

We also found that 1, 21, 15, and 5 MAT positive serum samples were negative by ELISA when using rLipL21, rLoa22, rLipL32 and rLigACon4-8 as antigens, respectively. However, Western blot analysis indicated only one of these ELISA negative samples was negative to all four recombinant antigens while the rest were positive to at least one of these antigens (Table 7).

In conclusion, the ELISA developed utilizing rLipL21, rLoa22, rLipL32 and rLigACon4-8 as antigens could increase the sensitivity and specificity of the ELISA test to detect leptospirosis in dogs. This ELISA test may be able to replace or supplement the current canine MAT test for the diagnosis of canine leptospirosis in the near future after further validation with more defined canine serum samples from known infected and vaccinated dogs.

## References

[1] Palaniappan RU, Ramanujam S, Chang YF (2007) Leptospirosis: pathogenesis, immunity, and diagnosis. Curr Opin Infect Dis 20:284–292. 17471039 10.1097/QCO.0b013e32814a5729

[2] Levett PN (2001) Leptospirosis. Clin Microbiol Rev 14:296–326. 11292640 10.1128/CMR.14.2.296-326.2001 PMC88975

[3] Sykes JE, Hartmann K, Lunn KF, Moore GE, Stoddard RA, et al (2011) 2010 ACVIM small animal consensus statement on leptospirosis: diagnosis, epidemiology, treatment, and prevention. J Vet Intern Med 25:1–13. 21155890 10.1111/j.1939-1676.2010.0654.x PMC3040842

[4] Phraisuwan P, Whitney EA, Tharmaphornpilas P, Guharat S, Thongkamsamut S, et al (2002) Leptospirosis: skin wounds and control strategies, Thailand, 1999. Emerg Infect Dis 8:1455–1459. 12498663 10.3201/eid0812.020180 PMC2738501

[5] Bharti AR, Nally JE, Ricaldi JN, Matthias MA, Diaz MM, et al (2003) Leptospirosis: a zoonotic disease of global importance. Lancet Infect Dis 3:757–771. 14652202 10.1016/s1473-3099(03)00830-2

[6] Cann KF, Thomas DR, Salmon RL, Wyn-Jones AP, Kay D (2013) Extreme water-related weather events and waterborne disease. Epidemiol Infect 141:671–686. 22877498 10.1017/S0950268812001653 PMC3594835

[7] Klopfleisch R, Kohn B, Plog S, Weingart C, Nockler K, et al (2010) An emerging pulmonary haemorrhagic syndrome in dogs: similar to the human leptospiral pulmonary haemorrhagic syndrome? Vet Med Int 2010:928541. 21274452 10.4061/2010/928541 PMC3025382

[8] Greenlee JJ, Bolin CA, Alt DP, Cheville NF, Andreasen CB (2004) Clinical and pathologic comparison of acute leptospirosis in dogs caused by two strains of *Leptospira kirschneri* serovar Grippotyphosa. Am J Vet Res 65:1100–1107. 15334844 10.2460/ajvr.2004.65.1100

[9] Greenlee JJ, Alt DP, Bolin CA, Zuerner RL, Andreasen CB (2005) Experimental canine leptospirosis caused by *Leptospira interrogans* serovars Pomona and Bratislava. Am J Vet Res 66:1816–1822. 16273916 10.2460/ajvr.2005.66.1816

[10] Gautam R, Wu CC, Guptill LF, Potter A, Moore GE (2010) Detection of antibodies against *Leptospira* serovars via microscopic agglutination tests in dogs in the United States, 2000–2007. J Am Vet Med Assoc 237:293–298. 20673110 10.2460/javma.237.3.293

[11] Birnbaum N, Barr SC, Center SA, Schermerhorn T, Randolph JF, et al (1998) Naturally acquired leptospirosis in 36 dogs: serological and clinicopathological features. J Small Anim Pract 39:231–236. 9631358 10.1111/j.1748-5827.1998.tb03640.x

[12] Hennebelle JH, Sykes JE, Carpenter TE, Foley J (2013) Spatial and temporal patterns of *Leptospira* infection in dogs from northern California: 67 cases (2001–2010). J Am Vet Med Assoc 242:941–947. 23517206 10.2460/javma.242.7.941

[13] Tangeman LE, Littman MP (2013) Clinicopathologic and atypical features of naturally occurring leptospirosis in dogs: 51 cases (2000-2010). J Am Vet Med Assoc 243:1316–1322. 24134583 10.2460/javma.243.9.1316

[14] Klaasen HL, van der Veen M, Molkenboer MJ, Sutton D (2013) A novel tetravalent *Leptospira* bacterin protects against infection and shedding following challenge in dogs. Vet Rec 172:181. 23180149 10.1136/vr.101100 PMC3582088

[15] Midence JN, Leutenegger CM, Chandler AM, Goldstein RE (2012) Effects of recent leptospira vaccination on whole blood real-time PCR testing in healthy client-owned dogs. J Vet Intern Med 26:149–152. 22182214 10.1111/j.1939-1676.2011.00852.x

[16] Mayer-Scholl A, Luge E, Draeger A, Nockler K, Kohn B (2013) Distribution of Leptospira serogroups in dogs from Berlin, Germany. Vector Borne Zoonotic Dis 13:200–202. 23428087 10.1089/vbz.2012.1121

[17] Thaipadungpanit J, Wuthiekanun V, Chantratita N, Yimsamran S, Amornchai P, et al (2013) Leptospira species in floodwater during the 2011 floods in the Bangkok Metropolitan Region, Thailand. Am J Trop Med Hyg 89:794–796. 24002484 10.4269/ajtmh.13-0124 PMC3795115

[18] Thaipadungpanit J, Wuthiekanun V, Chierakul W, Smythe LD, Petkanchanapong W, et al (2007) A dominant clone of Leptospira interrogans associated with an outbreak of human leptospirosis in Thailand. PLoS neglected tropical diseases 1:e56. 17989782 10.1371/journal.pntd.0000056 PMC2041815

[19] Ellis WA (2010) Control of canine leptospirosis in Europe: time for a change? Vet Rec 167:602–605. 21257439 10.1136/vr.c4965

[20] Gautam R, Guptill LF, Wu CC, Potter A, Moore GE (2010) Spatial and spatio-temporal clustering of overall and serovar-specific *Leptospira* microscopic agglutination test (MAT) seropositivity among dogs in the United States from 2000 through 2007. Prev Vet Med 96:122–131. 20580454 10.1016/j.prevetmed.2010.05.017

[21] Stokes JE, Kaneene JB, Schall WD, Kruger JM, Miller R, et al (2007) Prevalence of serum antibodies against six *Leptospira* serovars in healthy dogs. J Am Vet Med Assoc 230:1657–1664. 17542733 10.2460/javma.230.11.1657

[22] Suepaul SM, Carrington CV, Campbell M, Borde G, Adesiyun AA (2010) Serovars of *Leptospira* isolated from dogs and rodents. Epidemiol Infect 138:1059–1070. 19811697 10.1017/S0950268809990902

[23] Ye C, Yan W, McDonough PL, McDonough SP, Mohamed H, et al (2014) Serodiagnosis of equine leptospirosis by enzyme-linked immunosorbent assay using four recombinant protein markers. Clin Vaccine Immunol 21:478–483. 24451330 10.1128/CVI.00649-13 PMC3993115

[24] Cullen PA, Haake DA, Bulach DM, Zuerner RL, Adler B (2003) LipL21 is a novel surface-exposed lipoprotein of pathogenic *Leptospira* species. Infect Immun 71:2414–2421. 12704111 10.1128/IAI.71.5.2414-2421.2003 PMC153295

[25] Ristow P, Bourhy P, da Cruz McBride FW, Figueira CP, Huerre M, et al (2007) The OmpA-like protein Loa22 is essential for leptospiral virulence. PLoS Pathog 3:e97. 17630832 10.1371/journal.ppat.0030097 PMC1914066

[26] Pinne M, Haake DA (2013) LipL32 Is a Subsurface Lipoprotein of *Leptospira interrogans*: presentation of new data and reevaluation of previous studies. PloS one 8:e51025. 23323152 10.1371/journal.pone.0051025 PMC3544172

[27] Lin YP, Greenwood A, Nicholson LK, Sharma Y, McDonough SP, et al (2009) Fibronectin binds to and induces conformational change in a disordered region of leptospiral immunoglobulin-like protein B. J Biol Chem. 284:23547–23557. 10.1074/jbc.M109.031369 PMC2749129 19581300

[28] Palaniappan RU, Chang YF, Jusuf SS, Artiushin S, Timoney JF, et al (2002) Cloning and molecular characterization of an immunogenic LigA protein of *Leptospira interrogans* . Infect Immun 70:5924–5930. 12379666 10.1128/IAI.70.11.5924-5930.2002 PMC130282

[29] Fraune CK, Schweighauser A, Francey T (2013) Evaluation of the diagnostic value of serologic microagglutination testing and a polymerase chain reaction assay for diagnosis of acute leptospirosis in dogs in a referral center. J Am Vet Med Assoc 242:1373–1380. 23634681 10.2460/javma.242.10.1373

[30] Yan W, Saleem MH, McDonough P, McDonough SP, Divers TJ, et al (2013) Development of an enzyme-linked immunosorbent assay using a recombinant LigA fragment comprising repeat domains 4 to 7.5 as an antigen for diagnosis of equine leptospirosis. Clin Vaccine Immunol 20:1143–1149. 23720368 10.1128/CVI.00245-13 PMC3754523

[31] Chalayon P, Chanket P, Boonchawalit T, Chattanadee S, Srimanote P, et al (2011) Leptospirosis serodiagnosis by ELISA based on recombinant outer membrane protein. Trans R Soc Trop Med Hyg 105:289–297. 21353274 10.1016/j.trstmh.2011.01.008

[32] Palaniappan RU, Chang YF, Hassan F, McDonough SP, Pough M, et al (2004) Expression of leptospiral immunoglobulin-like protein by *Leptospira interrogans* and evaluation of its diagnostic potential in a kinetic ELISA. J Med Microbiol 53:975–984. 15358819 10.1099/jmm.0.45568-0

[33] Schreier S, Doungchawee G, Chadsuthi S, Triampo D, Triampo W (2013) Leptospirosis: current situation and trends of specific laboratory tests. Expert Rev Clin Immunol 9:263–280. 23445200 10.1586/eci.12.110

[34] Budihal SV, Perwez K (2014) Leptospirosis diagnosis: competancy of various laboratory tests. J Clin Diagn Res 8:199–202. 10.7860/JCDR/2014/6593.3950 PMC3939550 24596774

[35] Chen HW, Zhang Z, Halsey ES, Guevara C, Canal E, et al (2013) Detection of *Leptospira*-specific antibodies using a recombinant antigen-based enzyme-linked immunosorbent assay. Am J Trop Med Hyg 89:1088–1094. 24166046 10.4269/ajtmh.13-0041 PMC3854885

[36] Bourhy P, Vray M, Picardeau M (2013) Evaluation of an in-house ELISA using the intermediate species *Leptospira fainei* for diagnosis of leptospirosis. J Med Microbiol 62:822–827. 23493028 10.1099/jmm.0.054304-0

[37] Bomfim MR, Ko A, Koury MC (2005) Evaluation of the recombinant LipL32 in enzyme-linked immunosorbent assay for the serodiagnosis of bovine leptospirosis. Vet Microbiol 109:89–94. 15950404 10.1016/j.vetmic.2005.05.002

[38] Flannery B, Costa D, Carvalho FP, Guerreiro H, Matsunaga J, et al (2001) Evaluation of recombinant Leptospira antigen-based enzyme-linked immunosorbent assays for the serodiagnosis of leptospirosis. J Clin Microbiol 39:3303–3310. 11526167 10.1128/JCM.39.9.3303-3310.2001 PMC88335

[39] Croda J, Ramos JG, Matsunaga J, Queiroz A, Homma A, et al (2007) *Leptospira* immunoglobulin-like proteins as a serodiagnostic marker for acute leptospirosis. J Clin Microbiol 45:1528–1534. 17360842 10.1128/JCM.02344-06 PMC1865864

[40] Hartleben CP, Leal FM, Monte LG, Hartwig DD, Seixas FK, et al (2013) Serological analysis by enzyme-linked immunosorbent assay using recombinant antigen LipL32 for the diagnosis of swine leptospirosis. Curr Microbiol 66:106–109. 23064970 10.1007/s00284-012-0237-x

[41] Joseph S, Thomas N, Thangapandian E, Singh VP, Verma R, et al (2012) Evaluation and comparison of native and recombinant LipL21 protein-based ELISAs for diagnosis of bovine leptospirosis. J Vet Sci 13:99–101. 22437542 10.4142/jvs.2012.13.1.99 PMC3317464

[42] Oliveira TR, Longhi MT, de Morais ZM, Romero EC, Blanco RM, et al (2008) Evaluation of leptospiral recombinant antigens MPL17 and MPL21 for serological diagnosis of leptospirosis by enzyme-linked immunosorbent assays. Clin Vaccine Immunol 15:1715–1722. 18799647 10.1128/CVI.00214-08 PMC2583518

[43] Sankar S, Harshan HM, Somarajan SR, Srivastava SK (2010) Evaluation of a recombinant LigB protein of *Leptospira interrogans* serovar Canicola in an enzyme-linked immunosorbent assay for the serodiagnosis of bovine leptospirosis. Res Vet Sci 88:375–378. 20022618 10.1016/j.rvsc.2009.11.004

[44] Srimanote P, Wongdeethai N, Jieanampunkul P, Samonkiert S, Leepiyasakulchai C, et al (2008) Recombinant ligA for leptospirosis diagnosis and ligA among the *Leptospira* spp. clinical isolates. J Microbiol Methods 72:73–81. 18079011 10.1016/j.mimet.2007.10.012

[45] Nabity SA, Ribeiro GS, Aquino CL, Takahashi D, Damiao AO, et al (2012) Accuracy of a dual path platform (DPP) assay for the rapid point-of-care diagnosis of human leptospirosis. PLoS Negl Trop Dis 6:e1878. 23133686 10.1371/journal.pntd.0001878 PMC3486890

[46] Donskey CJ, Sunkesula VC, Jencson AL, Stone ND, Gould CV, et al (2014) Utility of a commercial PCR assay and a clinical prediction rule for detection of toxigenic Clostridium difficile in asymptomatic carriers. Journal of clinical microbiology 52:315–318. 24153132 10.1128/JCM.01852-13 PMC3911416

[47] Faine S, Adher B, Bloin C, Perolat P (1999) *Leptospira* and leptospirosis, 2nd e. MedSci. Medbourne, Australia.

[48] Group LBER Zoonoses and veterinary public health. In: Organization WH, editor.

[49] Public Health Surveillance and Informatics Program Office CfDCaP (2013) Leptospirosis (*Leptospira interrogans*).

[50] Raghavan R, Brenner K, Higgins J, Van der Merwe D, Harkin KR (2011) Evaluations of land cover risk factors for canine leptospirosis: 94 cases (2002–2009). Prev Vet Med 101:241–249. 21724280 10.1016/j.prevetmed.2011.05.010

[51] Barmettler R, Schweighauser A, Bigler S, Grooters AM, Francey T (2011) Assessment of exposure to *Leptospira* serovars in veterinary staff and dog owners in contact with infected dogs. J Am Vet Med Assoc 238:183–188. 21235371 10.2460/javma.238.2.183

[52] Andre-Fontaine G (2013) Diagnosis algorithm for leptospirosis in dogs: disease and vaccination effects on the serological results. Vet Rec 172:502. 23525483 10.1136/vr.101333

[53] Haake DA, Chao G, Zuerner RL, Barnett JK, Barnett D, et al (2000) The leptospiral major outer membrane protein LipL32 is a lipoprotein expressed during mammalian infection. Infect Immun 68:2276–2285. 10722630 10.1128/iai.68.4.2276-2285.2000 PMC97414

[54] Matsunaga J, Barocchi MA, Croda J, Young TA, Sanchez Y, et al (2003) Pathogenic *Leptospira* species express surface-exposed proteins belonging to the bacterial immunoglobulin superfamily. Mol Microbiol 49:929–945. 12890019 10.1046/j.1365-2958.2003.03619.x PMC1237129

[55] van Den Broek AHM, Thrusfield MV, Dobie GR, Willis WA (1991) A serological and bacteriolgical survey of leptospiral infeciton in dogs in Edinburg and Glasgow. J Small Animal Pract 32:118–124.

